# Family Structure, Resilience, and Academic Performance Among Emirati Medical Students: A Cross-Sectional Study from the United Arab Emirates

**DOI:** 10.1192/bjo.2026.11066

**Published:** 2026-06-30

**Authors:** Syed Fahad Javaid, Fatima Alnuaimi, Salma Alketbi, Gabriel Andrade, Fadwa Al Mugaddam

**Affiliations:** 1 Department of Psychiatry, College of Medicine and Health Sciences, United Arab Emirates University, Al Ain, UAE; 2 College of Medicine and Health Sciences, United Arab Emirates University, Al Ain, UAE; 3 Ajman University, Ajman, UAE

## Abstract

**Aims:**

Academic performance in medical school is shaped by socio-demographic and psychological determinants, yet evidence from Emirati cohorts remains scarce. This study examined associations between socio-demographic characteristics, psychological resilience and academic performance among Emirati undergraduate medical students, with a focus on family structure.

**Methods:**

We conducted a cross-sectional, questionnaire-based study among Emirati Medical students enrolled at the United Arab Emirates University. Undergraduate medical students were invited via institutional channels. The online survey captured self-reported cumulative Grade Point Average (GPA), socio-demographic data (age, gender, academic year, school type, high-school tutoring, family structure, curriculum type) and psychological resilience using the Brief Resilience Scale (BRS). Descriptive statistics summarised the sample. Group differences in GPA were tested using independent-samples t-tests and one-way ANOVA, and the association between GPA and resilience was examined with Spearman correlation, withsignificance set at p<0.05. A total of 115 complete responses were analysed.

**Results:**

Respondents had a mean age of 20.3 years (SD 1.23); 75.7% were female. Slightly more attended public than private UAE schools (52.2% vs 47.8%); 71.3% had not received private tutoring. Most were in years 3–4 (64.4%). The majority lived in nuclear families (82.6%), with 12.2% in extended families and 5.2% in single-parent/own-household arrangements; 48.7% followed a UAE high-school curriculum and 51.3% non-UAE curricula. Mean cumulative GPA was 3.45 (SD 0.37). Resilience scores indicated moderate resilience (mean total 19.1, SD 3.66; mean item score 3.18, SD 0.61) and were not associated with GPA (Spearman r=0.022, p=0.814). There were no significant GPA differences by gender, tutoring, school type or curriculum (all p>0.05). Family structure showed a significant effect (F(2,112)=3.14, p=0.047), with students in single-parent/own-household arrangements having a lower GPA (M=3.09, SD 0.45) than those in nuclear families (M=3.46, SD 0.37), while those in extended families had a similarly high GPA.

**Conclusion:**

In this cohort of Emirati medical students, academic performance was generally high and similar across gender, resilience, school type, tutoring, or curriculum. However, lower GPA among students living in single-parent or independent households suggests that reduced or disrupted family support may affect outcomes. Routine screening for social and family stressors, along with targeted support for students with less stable family arrangements, may help mitigate emerging inequalities.

No financial sponsorship has been received for this study.

